# Malingering as an Actual Risk

**Published:** 1913-05-03

**Authors:** 


### OUR NATIONAL INSURANCE ACT DEPARTMENT.

### LI.—Malingering as an Actual Risk.

One of the dangers to which the financial success of the National Insurance Act is exposed is that of malingering. This was, of course, realised at the outset, but it was hoped that sufficient safeguards had been imposed in the Act itself and in the regulations of the Insurance Commissioners to prevent any serious ill-effects from this source. It is as yet somewhat early to say definitely to what extent malingering is actually happening, but that certain of the claims for sickness benefit which have been made on the approved societies have not really been justified seems to be agreed by the officials of several of the most important societies. The matter was considered at the meeting of the Faculty of Insurance, and several suggestions were made with a view to protecting the funds of the societies. The difficulty at the moment is to know how best to prevent such claims being made, and how to detect them from the mass of *bona-fide* claims when actually received.

Friendly society experience in the past has shown that the rate of claim for sick pay has varied directly with the size of the society, or inversely with the efficiency of supervision of claims. Small societies, and small lodges or courts of the large affiliated orders, in which the members have had a personal interest in the management, have always shown a lighter rate of sickness claim than similarly constituted societies with large numbers of members, many of whom have had little or no interest in the management. The reasons for this are twofold. In the first place, it is probable that the small societies were more careful in admitting members to see that only such persons as could show a good health record were actually admitted, and further that to some extent, at least, the moral character of applicants was closely scrutinised. The second reason is probably of equal importance—namely, that in the smaller societies certain of the members were elected as stewards or sick visitors, and it devolved on these members to visit everyone in receipt of sick pay at least once a week, and to report to the committee of management in case all the rules of the society were not being observed.

Now the approved societies have been fashioned on the plan of the old friendly societies, but in the two matters just dealt with the resemblance is but small. Even the smallest of the approved societies seemed most anxious to obtain a large membership, and the result has been that little attention has been paid either to past health records or to moral character in admitting applicants to membership. Again, the requirements of the Insurance Commissioners have practically necessitated the concentration of the management in the hands of the secretary and principal officers, and as the members' own contributions are not more than four-ninths of the benefits they have not felt the same necessity for supervision as when they paid the whole of the contribution themselves. Furthermore, as we pointed out last week, considerable difficulty has been experienced in restricting the expenses to the allowance prescribed by the Insurance Commissioners, and in the circumstances it is hardly to be wondered at that expenditure on sick visitors or inspectors has not been incurred.

All these features of the constitution of the approved societies have tended to a certain laxity in the supervision of claims, and in consequence a high rate of sickness may be expected to be shown unless matters can speedily be rectified. A somewhat disquieting fact from the point of view of the societies is the apparent facility with which many members have been able to obtain certificates of incapacity from their panel doctors. It will be remembered that in consideration of the increased grant to the doctors the certificates required for the purpose of claiming sickness benefit were to be supplied without charge. It may be that the readiness with which many of the certificates have been given is not entirely unconnected with the dislike of the doctors to the conditions of their panel service. If this be in fact the case, it is only another evidence of the short-sighted policy of the Chancellor of the Exchequer in his dealings with the medical profession. It is, of course, a serious imputation that certificates are being furnished without justification, and we do not suggest that this is being done except in rare instances. That there have been cases, however, in which certificates have been signed by panel doctors when a refusal should have been made seems to be agreed by society officials.

Sir J. Collie, medical officer of the London County Council, speaking at the meeting of the Faculty of Insurance, very much doubted whether the present method of medical and lay control was sufficient to prevent inroads on the funds of the societies. He suggested the appointment of medical referees to meet the difficulty, but this would require further expenditure, which the societies are at present unable to bear.

Apart from specific instances that have come to our notice of claims unduly protracted, the principal evidence that malingering is taking place lies in the fact that so few claims are made for less than a week's sickness benefit. For the first three days of incapacity for work no sickness benefit is payable. Hence, if a claim for a week's sick pay is made, it is implied that the actual illness has lasted ten days; and it is a curious fact that so large a majority of the claims should be for an exact week. It is possible, of course, that a number of insured persons are as yet unaware that sickness benefit accrues from day to day, and that a claim for benefit would be paid if only for a single day, provided the sickness lasted over the fourth day. There are also probably a number of insured persons who would not think it worth while to apply for sickness benefit unless they could claim a week's payment. Both these causes would operate to throw into relief the number of claims for an exact week's benefit, but it is hardly possible that they can entirely account for the feature referred to, which, we understand, is being experienced by most of the large societies.

If it, indeed, be a fact that a considerable number of insured persons are not claiming benefit when actually entitled to it, there would be added ground for uneasiness, for we learn that during the past few weeks the proportion of members on the funds of one of the largest societies has been about 3 per cent. of the total membership, and this is a high figure when it is remembered that at the present time there are no cases of disablement benefit.

In the long run malingering will not pay, for those who remain longest on the funds at full sickness benefit rates will find that they will be the soonest to reach the period when only disablement benefit—or reduced sick pay—can be claimed. Thus in the end it is possible that matters will adjust themselves automatically, but in the meantime moral harm will be done unless more effective measures are taken to repress all but absolutely *bona-fide* claims.

